# Role of UBE2C in Brain Cancer Invasion and Dissemination

**DOI:** 10.3390/ijms242115792

**Published:** 2023-10-31

**Authors:** Stefani Domentean, Eunice Paisana, Rita Cascão, Claudia C. Faria

**Affiliations:** 1 Instituto de Medicina Molecular João Lobo Antunes, Faculdade de Medicina da Universidade de Lisboa, Edifício Egas Moniz, Av. Prof. Egas Moniz, 1649-028 Lisboa, Portugal; stefani.domentean@medicina.ulisboa.pt (S.D.); epaisana@medicina.ulisboa.pt (E.P.); ritacascao@medicina.ulisboa.pt (R.C.); 2 Department of Neurosurgery, Hospital de Santa Maria, Centro Hospitalar Universitário Lisboa Norte (CHULN), Av. Prof. Egas Moniz, 1649-028 Lisboa, Portugal; 3 Clínica Universitária de Neurocirurgia, Faculdade de Medicina da Universidade de Lisboa, Av. Prof. Egas Moniz, 1649-028 Lisboa, Portugal

**Keywords:** brain tumors, glioblastoma (GB), brain metastases (BM), ubiquitin-conjugating enzyme E2C (UBE2C), invasion, dissemination, clinical biomarker, targeted therapies, prognosis

## Abstract

Glioblastoma (GB) and brain metastases (BM) are the most common brain tumors in adults and are invariably associated with a dismal outcome. These highly malignant tumors share common features including increased invasion and migration of the primary or metastatic brain cancer cells, whose triggering mechanisms are largely unknown. Emerging evidence has suggested that the ubiquitin-conjugating enzyme E2C (UBE2C), essential for controlling cell cycle progression, is overexpressed in diverse malignancies, including brain cancer. This review highlights the crucial role of UBE2C in brain tumorigenesis and its association with higher proliferative phenotype and histopathological grade, with autophagy and apoptosis suppression, epithelial-to-mesenchymal transition (EMT), invasion, migration, and dissemination. High expression of UBE2C has been associated with patients’ poor prognosis and drug resistance. UBE2C has also been proven as a promising therapeutic target, despite the lack of specific inhibitors. Thus, there is a need to further explore the role of UBE2C in malignant brain cancer and to develop effective targeted therapies for patients with this deadly disease.

## 1. Introduction

The most common brain tumors in the adult population include glioblastoma (GB), a primary brain tumor arising from cells within the brain, and brain metastases (BM), secondary lesions that arise from systemic cancers that disseminate to the brain during disease progression. Even though brain tumors are rare, accounting for 3% of all cancer types, patients have increased mortality and severe disabilities [1,2]. Standard of care treatment includes neurosurgical resection, radiation therapy, and chemotherapy. Nevertheless, primary and secondary brain tumors are challenging to treat since they may not be amenable to surgical resection due to their location in eloquent areas or to the number of lesions and due to the blood–brain barrier (BBB), which may be a limiting factor in the efficacy of systemic chemotherapies [2]. Furthermore, no new and more effective therapies have been found in the past years to treat brain tumors [3]. Recently, immunotherapy has shown promising results for patients with metastatic brain lesions from melanoma and lung cancer [4,5,6]. However, the overall survival of patients with malignant brain tumors, particularly GB and BM, remains poor despite decades of research and clinical trial development [3].

Glioblastoma is the most frequently diagnosed malignant primary brain tumor in adults. It is a grade 4 tumor according to the classification of the World Health Organization (WHO) and the most aggressive type of glioma [7]. Currently, treatment includes maximal safe surgical resection followed by radiation therapy and chemotherapy with temozolomide. Nonetheless, the median survival of treated patients is approximately 15 months, and they usually relapse in a short period of time with a poorer prognosis, exhibiting a progression-free survival of 1.5–6 months and an overall survival of 2–9 months. Therefore, despite all therapeutic developments, there are currently no effective therapies for GB [3,8].

Invasion is a main feature of GB through the infiltration of cancer cells in the brain parenchyma, leading to therapeutic resistance and tumor recurrence [9,10,11]. Glioma cells are histologically similar to glial progenitor cells that have proliferative, migratory, and dedifferentiation capacities, suggesting that mechanisms involved in the migration of neuroepithelial cells during embryonic development and regenerative processes are also involved in GB invasion [10,12]. This process comprises the detachment of cancer cells from the main tumor, degradation of the extracellular matrix, and migration. Studies have reported that the epithelial-to-mesenchymal transition (EMT) plays a critical role in GB progression, particularly in migration and invasion [10,13]. Moreover, glioma stem cells (GSCs), a population that exhibits several characteristics of both neural progenitor and stem cells, have been described as responsible for tumor invasion, possibly through the WNT, TGF-β, HGF/MET, PI3K/AKT, and STAT3 signaling pathways [9]. Ephrin receptors, Rho GTPases, and CK2 have also been proposed as molecules that interfere with GB invasiveness [9]. However, the mechanisms responsible for GB invasion are largely unknown. Most therapeutic strategies are directed at the highly proliferative tumor mass, whereas there are no anti-invasion molecules approved so far for clinical purposes [11,14].

Another common brain tumor in the adult population is BM. BM arise from the dissemination of cancer cells from a systemic primary tumor to the brain and are estimated to be 10 times more frequent than primary brain tumors. Additionally, the incidence of BM is thought to be increasing, possibly as a result of the improvement in diagnostic methods and better treatment of systemic cancer [15,16]. Approximately 20–30% of cancer patients develop BM during disease progression, with brain metastatic disease being the main cause of morbidity and mortality [17]. The most common primary malignancies that disseminate to the brain include lung (40–50%), breast (15–20%), melanoma (5–10%), and gastrointestinal (4–6%) cancers [17]. At the time of BM diagnosis, about 37–50% of patients exhibit a single brain lesion, while 50–63% have multiple BM, making it more difficult to treat [17,18,19]. Standard of care treatment of BM includes surgery, radiotherapy, and chemotherapy. Nonetheless, patients have a poor prognosis and about 60% develop local recurrence within 1 year [20,21]. Surgery is limited to patients with few symptomatic lesions in accessible brain locations and with a good Karnofsky performance status [15,18]. Chemotherapeutic efficacy in the treatment of BM has been difficult to determine. Most clinical trials of systemic treatments exclude BM patients, and response rates reveal the efficacy in the primary tumor but not the outcome in the brain lesions [22]. 

Dissemination is a complex multi-step process that comprises genetic, epigenetic, and biological alterations of cancer cells. This metastatic cascade starts with the migration and invasion of cancer cells into adjacent tissues, followed by intravasation into the bloodstream and colonization of a new secondary site [23]. Recently, it was shown that the underlying cause for local recurrence and consequent worse patient survival was the capacity of some brain metastatic cancer cells to undergo a reprogramming process and acquire features that allow the invasion of adjacent brain tissue [20]. Additionally, it has been reported that BM from different primary tumor origins share mutations in the PI3K/ATK/mTOR signaling pathway and mutations associated with sensitivity to PI3K, CDK, HER2/EGFR, and MAPK inhibitors [24]. These pathways are known to be associated with tumorigenesis, proliferation, apoptosis, EMT, invasion, metastasis, stem-like behavior, and drug resistance in metastatic cancer cells, including BM [16,25,26]. Moreover, *MYC*, *YAP1*, and *MMP13* genes were amplified in BM in comparison with their corresponding primary lung tumors, suggesting that these genes may be driving BM [24]. Other genes were reported as contributing to BM development, namely *p53*, *KRAS*, *DSC2*, *WNT–TCF*, *HB–EGF*, *COX2*, *BMP-2*, src, serpins, and cathepsin-S [27,28,29,30,31,32].

Despite previous attempts to study the mechanisms that underlie the invasion and dissemination of cancer cells to the brain and within the brain, these remain poorly understood. Uncovering these mechanisms is an unmet medical need critical for the development of novel therapeutic approaches that can improve patients’ survival and quality of life.

The ubiquitin system has been gaining significant relevance in cancer research due to its role in several cellular processes. In the last years, growing data have been exhibiting the human ubiquitin-conjugating enzyme E2C (UBE2C) as a relevant player in tumorigenesis and a valuable biomarker in several types of cancers. In this review, we aim to discuss the role of UBE2C in brain cancer invasion and dissemination and its potential therapeutic targeting in brain tumor patients.

## 2. UBE2C

### 2.1. Ubiquitin Proteasome System

Damaged proteins, either caused by intrinsic or extrinsic factors, affect different cell functions, and consequently cell viability. The ubiquitin proteasome system (UPS) is responsible for eradicating these damaged proteins and maintaining protein homeostasis. Additionally, the UPS controls the concentrations of cell cycle regulators according to the cell phase, regulating cell cycle progression [33,34].

Protein degradation starts with the ubiquitination of damaged proteins, an ATP-dependent process that requires three types of enzymes, namely ubiquitin-activating enzyme (E1), the ubiquitin-conjugating enzyme (E2), and ubiquitin ligases (E3), which operate consecutively in a cascade. The E1 enzyme activates the ubiquitin molecule through ATP hydrolyzation followed by adenylation of the glycine residue present in the C-terminal of ubiquitin and its linkage to the cysteine active site of E1. Subsequently, ubiquitin is transferred to the E2 enzyme, allowing the conjugation between E2 and E3, which in turn recognizes and binds the target protein while catalyzing the ubiquitin transfer to the latter. These enzymes operate in a methodical manner to polyubiquitinate the substrates so that these can be recognized and degraded by the proteasome [33,34].

Dysregulation of the UPS leads to an accumulation of damaged proteins that have key roles in DNA damage repair, cellular differentiation, cell cycle control, apoptosis, and other vital functions. This dysregulation has been associated with several diseases, namely neurodegenerative disorders and different types of cancer, including brain tumors [35]. Interestingly, a high-throughput small interfering RNA (siRNA) screen in T98G glioma cells reported that 22% of the genes identified as being crucial for GB survival encoded for components of the proteasome subunits 20S and 26S [36]. Moreover, aberrant levels of different E2 and E3 enzymes have been reported in glioma, having either oncogenic or antitumoral effects [37,38,39,40]. On the other hand, altered levels of deubiquitinating enzymes (DUBs), which enhance protein stability by cleaving the ubiquitin and thus reversing ubiquitin-mediated proteolysis, can also affect crucial signaling pathways and consequently lead to malignancies. In particular, DUBs have been linked to the regulation of GSCs’ pluripotency and microenvironment, as well as radiation and chemotherapy resistance [41,42,43].

### 2.2. Physiological Role of UBE2C in Cell Cycle

The *UBE2C* gene (also known as *UBCH10*) is located at chromosome 20q13.12 and can generate 7 transcript variants, being the longest transcript translated into a protein composed of 179 amino acids with a total molecular weight of 19.65 kDa [44,45]. The UBE2C protein is a member of the E2 family and is responsible for the transfer of ubiquitin to the protein substrates targeting proteasome-mediated degradation [45]. The N-terminal of UBE2C is composed of the first 28 residues and allows protein recognition and conjugation, namely with the anaphase-promoting complex/cyclosome (APC/C). The E2 core domain contains a catalytic Cys114 in the active site that is responsible for the formation of an adduct with E1-activated ubiquitin through a thiol ester bond. Together with E3 ligases or independently, UBE2C then donates the ubiquitin to target proteins for proteasome degradation [44,45,46].

**Figure 1:**
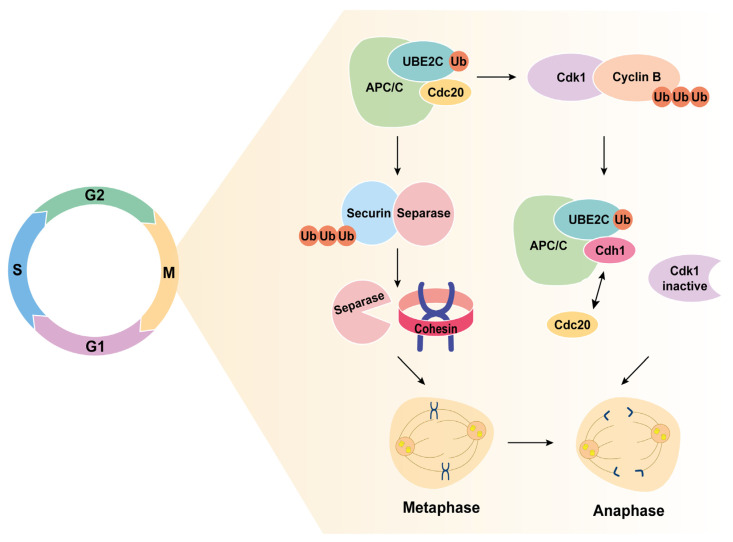
UBE2C induces metaphase to anaphase transition during the cell cycle. UBE2C-induced degradation of cyclin B inactivates Cdk1, allowing the exchange of Cdc20 by Cdh1 in the APC/C-UBE2C complex, which becomes activated. The activated complex degrades securin, allowing separase to be activated and degrade the cohesin rings of sister chromatids. This leads to anaphase onset and mitotic exit.

UBE2C is located in the nucleus and cytoplasm of cells, being scarcely expressed in normal tissues, at both RNA and protein levels [45]. UBE2C is involved in different steps of the cell cycle, controlling its progression (Figure 1). The APC/C is a multi-subunit complex that functions as an E3 ubiquitin ligase and interacts with UBE2C, inducing the metaphase/anaphase transition and the exit from mitosis [45,47]. Along with APC/C, UBE2C degrades securin that normally forms an inhibitory complex with separase. Separase is then activated and degrades the cohesin rings that link the two sister chromatids together, leading to chromatid separation and anaphase onset [45,48].

Additionally, UBE2C promotes the inactivation of the mitotic cell cycle spindle assembly checkpoint (SAC), necessary to guarantee the appropriate separation of the sister chromatids into two daughter cells, averting aneuploidy [45]. This is ensured by UBE2C-driven degradation of mitotic cyclin B, which forms a complex with cyclin-dependent kinase 1 (Cdk1), inactivating the latter. Cdk1 inactivation allows the replacement of Cdc20 by Cdh1 in the APC/C-UBE2C complex, promoting the sequential ubiquitination and degradation of key regulators. This will lead to mitotic exit and progression to G1 and S phases. Once UBE2C has fulfilled its function, it is degraded via autoubiquitination in association with the APC/CCdh1 and, therefore, its expression is controlled through a positive autoregulatory feedback loop dependent on APC/C substrate concentrations that compete with UBE2C. UBE2C levels increase gradually over S and G2 phases when cyclin A inactivates APC/C, allowing the re-accumulation of UBE2C [44,45,49,50].

*UBE2C* overexpression triggers whole chromosome instability and consequent missegregation and aneuploidy [51]. Its accumulation also stimulates cell proliferation, suggesting that *UBE2C* overexpression may play an important role in tumorigenesis and tumor progression [45].

### 2.3. UBE2C and Systemic Cancer

The overexpression of *UBE2C* has been described in many human cancers, namely breast, lung, thyroid, endometrial, renal, prostate, pancreatic, esophageal, hepatocellular, gastrointestinal, and brain tumors [47,52,53,54]. The overexpression of this gene has been associated with tumor progression and worse prognosis. Human cancers with higher levels of UBE2C exhibit increased aggressiveness, low differentiation, and metastatic predisposition and are associated with reduced patient survival [45,47,52,54]. Table 1 presents the data from the most relevant papers regarding the role and clinical impact of UBE2C expression on different types of malignancies. These studies were chosen based on information on chemotherapeutic resistance, signaling pathways, and mechanisms that shed light onto possible therapeutic strategies.

#### 2.3.1. Breast Cancer

Breast cancer is the most frequently diagnosed cancer, with an 11.7% global incidence, and is the leading cause of cancer death in women [79,80]. Up to one-third of breast cancer patients develop BM. UBE2C overexpression has been shown to have a prognostic value in breast cancer [47,55]. Recently, high levels of this protein have been identified as an independent prognostic marker in breast cancer patients, correlated with poor prognosis, high tumor grade, lymphovascular invasion, lymph node metastases, hormone receptor negativity, HER2 positivity, and stem-like features [56,57,81]. Invasive breast tumors seem to have a higher expression of UBE2C [82]. In contrast, *UBE2C* knockdown leads to inhibition of proliferation and invasion in breast cancer cells, as well as a decrease of p-AKT, p-mTOR, and hypoxia-inducible factor 1-α (HIF-1α) and an increase of phosphorylated phosphatase and p-PTEN levels. UBE2C is thought to promote breast cancer proliferation by activating the AKT/mTOR signaling pathway, a known key player in metastasis [58,83]. UBE2C is also associated with cell cycle-related biomarkers, such as p53, ki67, EGFR, and PI3K, and its overexpression reduces sensitivity to chemotherapy [59,60]. More recently, it has been reported that the circular RNA circ_0059457 is responsible for UBE2C-driven breast cancer cell migration, invasion, and metastasis through an miR-140-3p-dependent mechanism [84].

#### 2.3.2. Lung Cancer

Lung cancer is the second most common cancer, with a global incidence of 11.4%, and is the leading cause of cancer-related deaths. The five-year survival for patients with lung cancer is 10–20% [80]. One of the main concerns in lung cancer is the high incidence of metastasis to the brain [85]. UBE2C was identified as an independent prognostic factor associated with primary tumor size, lymph node metastases, Tumor/Node/Metastasis (TNM) stage, overall survival, and disease-free survival in lung cancer patients [62,86]. Particularly, in non-small cell lung carcinomas (NSCLC), *UBE2C* has a positive correlation with tumor grade, being overexpressed in poorly differentiated tumors [63,87]. The expression of UBE2C can be downregulated through the knockdown of *MALAT1*, leading to the inactivation of WNT, PI3K/AKT, and MAPK/ERK, thus inhibiting proliferation, migration, and invasion of lung cancer cells [64]. Additionally, UBE2C induces chemoresistance through the increased expression of the multi-drug resistance (*MDR1*) gene [63], *ABCG2*, and *ERCC1* [65]. In NSCLC cells, it was shown that UBE2C levels directly correlate with *p53* mutational status and inversely correlate with *EGFR* mutational status [66]. Moreover, UBE2C selectively represses autophagy, leading to enhanced cell proliferation and invasive tumor growth [88]. Recently, it has been reported that KrasG12D-induced lung tumorigenesis requires UBE2C expression, which couples with APC/C to promote ubiquitylation and degradation of DEP domain-containing mechanistic target of rapamycin (mTOR) interacting protein (DEPTOR), leading to activation of mTOR signaling [67].

#### 2.3.3. Other Neoplasms

UBE2C has been described as a promoter of migration and invasion of tumor cells in several other types of cancer [68,71,72,89,90]. In hepatocellular carcinoma, *UBE2C* upregulation is associated with tumor invasion, dedifferentiation, and poor prognosis [68]. On the other hand, the depletion of F-box protein 43 results in *UBE2C* downregulation, suppression of p53 proteasomal degradation, and inhibition of cell proliferation and invasion [91]. In hepatoblastoma clinical samples, high levels of UBE2C mRNA seem to increase distant metastasis and death rates. Moreover, the knockdown of *UBE2C* in the HuH6 hepatoblastoma cell line and in the HB-243 cell line from a patient-derived xenograft decreased cell viability by up to 44% and cell migration by 65% [92]. In osteosarcoma cells, *UBE2C* knockdown impairs invasion and migration [93]. This effect has also been demonstrated in renal, prostate, and gastric cancers [94,95,96]. Recently, Huang et al. proposed that patients with adrenocortical carcinoma with high UBE2C expression may present a worse prognosis by inducing self-renewal of adrenocortical stem cells [73].

An association between UBE2C and p53, a tumor suppressor protein involved in the G2/M checkpoint and apoptosis of cells with damaged DNA [97], is described in endometrial, hepatocellular, thyroid, and lung cancer cells, where UBE2C-induced p53 degradation promotes migration, invasion, and EMT [68,71,72,98].

The PI3K/AKT/mTOR/signaling pathway has been correlated with UBE2C levels in several cancers, including thyroid, gastric, and pancreatic cancer. In gastric and cervical cancer, *UBE2C* knockdown leads to increased apoptosis induced by cisplatin and reduced phosphorylation of ERK, AKT/PKB, and p38 [99,100]. In neck squamous cell carcinoma, UBE2C expression is correlated with an increase in HIF-1 α, leading to glycolysis initiation and migration [101]. Finally, the UBE2C protein network shows strong associations with cancer-related proteins involved in the formation and maintenance of the mitotic spindle, chromosome segregation, microtubule depolarization, centrosome integrity, and homologous recombination and repair of DNA in different cancers [52]. Recently, a strong association between UBE2C and stromal and immune score was described in clear renal cell carcinoma. The expression levels of UBE2C are significantly associated with extended levels of regulatory T cell infiltration, CD4+ memory T cells, and M0 macrophages. In addition, several immune checkpoint genes exhibit a positive correlation with *UBE2C* [74]. Other studies have reported a correlation between UBE2C and immune cell infiltration [61,69,72,73,102]. Specifically in hepatocellular carcinoma, UBE2C is positively correlated with infiltration of regulatory T cells and T follicular helper cells while presenting a negative correlation with macrophage infiltration [69]. On the other hand, in adrenocortical carcinoma, UBE2C expression is positively linked with T helper Th1 and Th2 cells and negatively correlated with regulatory T cells and M2 macrophage infiltration [73]. 

## 3. UBE2C and Brain Cancer Invasion and Dissemination

The role of UBE2C has been studied in brain tumors, including gliomas, meningiomas, and BM. UBE2C expression was analyzed in astrocytic tumors, and authors concluded that GB exhibits high levels of this protein compared with low-grade astrocytomas, indicating a correlation between UBE2C expression and glioma tumor grade. It was also shown that *UBE2C* overexpression is only found in cancer cells, and not in normal tissues [75,76,103,104]. Ma et al. described the impact of UBE2C expression on the outcome and overall survival of patients with GB, making it a potential biomarker [104]. Additionally, an association between UBE2C and the proliferative marker Ki-67 was described [103]. Furthermore, a functional interaction between UBE2C and p53 has been suggested in glioma cells, where p53 downregulation, possibly due to UBE2C-induced degradation, results in diminished apoptosis in these cells [77]. 

It has been shown that the Forkhead box transcription factor M1 (FoxM1) triggers *UBE2C* transcription by binding to its promoter regions and is associated with poor prognosis in gliomas [75]. FoxM1 has a key role in cell cycle progression, with a peak during S and G2/M phases, which temporally corresponds to a peak in UBE2C [105]. Interestingly, FoxM1 is upregulated in brain tumors, particularly GB and meningioma [75,106]. Upregulation of UBE2C is observed in GB tissues, mainly in the proneural subtype, characterized by neurogenesis-related gene expression and increased invasion [107,108]. The combined upregulation of UBE2C and aurora kinase B (AURKB), an important player in the cell cycle, is associated with a dismal outcome, therapeutic resistance, and reduced overall survival in glioma patients [76].

In meningiomas, *UBE2C* overexpression correlates with higher histological grade, increased proliferation, and poor prognosis [109]. When exploring *UBE2C* function in meningioma cells with different grades, it was reported that silencing this gene inhibits proliferation, migration, and invasion. Moreover, *UBE2C* knockdown induces apoptosis by increasing Bax and caspase-3 and by decreasing Bcl-2 levels [110]. It was also demonstrated that *RIZ1*, a tumor suppressor gene, is markedly decreased and regulates UBE2C in a c-Myc-dependent manner in malignant meningioma [110,111]. 

Recently, Paisana et al. reported the association of UBE2C and BM [78]. The authors have demonstrated that UBE2C is highly expressed in human BM from different primary tumors in comparison to normal tissues. In BM patients, high levels of UBE2C are associated with reduced survival, suggesting that it may be a useful clinical biomarker of prognosis in patients with brain metastatic disease. *UBE2C* overexpression increases migration and invasion of breast and lung cancer cells. In addition, mice orthotopically injected with *UBE2C*-overexpressing cancer cells develop increased leptomeningeal dissemination, an aggressive phenotype of brain metastatic disease [78]. Another recent study using single-cell RNA sequencing in human BM from diverse cancer types identified *UBE2C* as a signature gene in the proliferative molecular subgroup [112].

**Figure 2:**
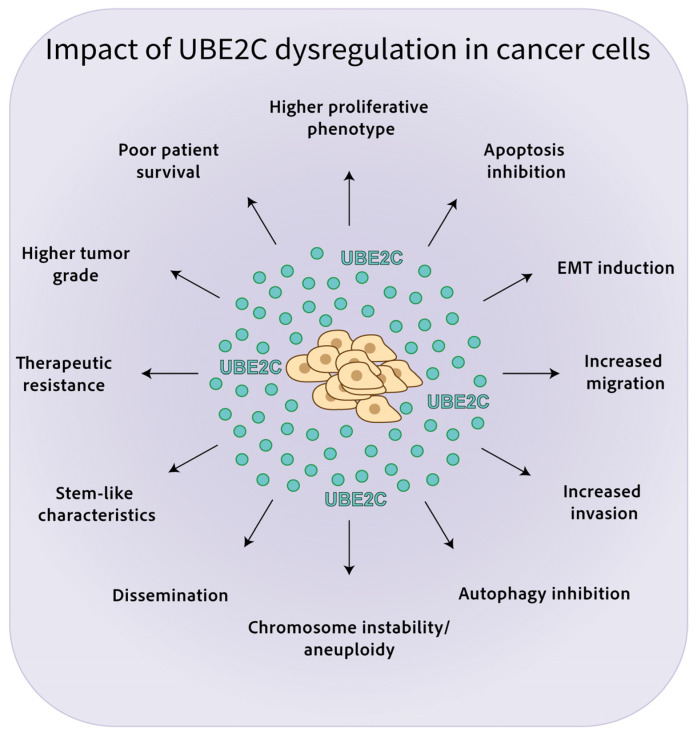
Impact of UBE2C dysregulation in cancer cells. High UBE2C levels are associated with chromosome instability/aneuploidy, increased proliferation, invasion, and migration, EMT, reduced apoptosis, autophagy inhibition, stem-like properties, dissemination, and therapy resistance, leading to poor patient survival in several malignancies.

The effects of UBE2C dysregulation in cancer are highlighted in Figure 2.

## 4. UBE2C Inhibition as a Potential Therapeutic Target

The best approach to target the UPS in order to achieve better selectivity with less toxicity is through inhibition of the E2 enzymes or E3 ligases, which have been reported as dysregulated in several cancers. E2 enzymes are abnormally translated in a variety of cancers, being responsible for tumor progression and unfavorable biological and clinical characteristics, which emphasizes the relevance of UBE2C as a potential therapeutic target. Until now, UBE2C targeting has been limited to experimental studies, making the development of UBE2C inhibitors essential for patient care [113,114].

*UBE2C* knockdown using interference RNA inhibits proliferation and promotes activation of the tumor suppressor p53 and apoptosis in U251 glioma cells [77]. It was also observed that *UBE2C* knockdown suppresses glioma cell growth by 40% in comparison to control cells and induces autophagy through inactivation of the PI3K/Akt/mTOR pathway [75]. 

In breast cancer cells, *UBE2C* silencing inhibits ERK and AKT phosphorylation and increases the levels of phosphorylated PTEN, which acts as a negative regulator of the PI3K/AKT pathway [58]. UBE2C inhibition sensitizes breast cancer cells to treatment with radiation and doxorubicin (alkylating agent), as well as to anti-hormonal agents such as tamoxifen (estrogen receptor modulator) and letrozole (nonsteroidal aromatase inhibitor) [60]. *UBE2C* depletion in triple-negative breast cancer cells decreases proliferation, which is substantially enhanced in the presence of paclitaxel, suggesting a possible new combination therapy [115]. Intriguingly, CDK4/6 inhibitors can suppress *UBE2C* expression in estrogen receptor-positive breast cancer cells [116]. 

In ovarian cancer, *UBE2C* silencing decreases cell proliferation in vitro and in vivo and results in G2/M arrest, increased cell apoptosis, and reversed cisplatin resistance. In addition, cyclin B1, CDK1, and Bcl-2 levels are downregulated, while Bax expression is upregulated [117]. In gastric cancer cells, *UBE2C* depletion impairs the ERK1/2 signaling pathway in vitro, while decreasing tumor volume in vivo. Additionally, the ERK1/2 inhibitor U0126 reduces ERK1/2 phosphorylation and reverses the effect of UBE2C on cell proliferation, migration, and invasion [96]. Similar results were observed in melanoma cells, where in vivo *UBE2C* knockdown suppresses the growth of xenografted tumors [118]. In hepatocellular carcinoma, *UBE2C* downregulation increases the sensitivity to chemotherapeutic agents including doxorubicin, 5-fluorouracil (cellular thymidylate synthase inhibitor), and the multikinase inhibitor sorafenib [70]. *UBE2C* silencing also sensitizes colorectal cancer cell lines to bortezomib (proteasome blocker) and oxaliplatin (alkylating agent), inducing apoptosis [119]. In thyroid carcinoma cells, *UBE2C* knockdown decreases cell proliferation, migration, and invasion, promotes apoptosis, and reduces cisplatin resistance [72]. UBE2C depletion in adrenocortical carcinoma cells suppresses cell invasion and migration by EMT inhibition, DNA damage repair, and induction of apoptosis [73].

Interestingly, the manipulation of miRNAs, namely miR661-3p and miR-381-3p, has shown the ability to target UBE2C in NSCLC and prostate cancer cells, respectively, leading to reduced proliferation, invasion, and metastasis [120,121].

CCI-779, an mTOR inhibitor, significantly decreases UBE2C in prostate cancer cell lines. CCI-779 inhibits the recruitment of androgen receptor-coactivator to the UBE2C enhancers and, consequently, reduces the loading of RNA polymerase II and transcription. In addition, CCI-779 inhibits cell proliferation and invasion in vitro and decreases tumor growth in mouse xenografts [122]. Moreover, docetaxel (microtubule depolymerization inhibitor) treatment in *UBE2C*-depleted prostate cancer cells reduces the degradation rate of cyclin B1, suggesting less ability to recover from the mitotic arrest induced by docetaxel. Therefore, UBE2C seems to be a key player in the regulation of mitotic slippage and a mediator in docetaxel resistance in prostate cancer [123]. Treatment of cervical cancer cells with vorinostat, a histone deacetylase inhibitor that reduces PI3K/AKT/mTOR signaling and UBE2C activation, reverses EMT through downregulation of N-cadherin and vimentin and upregulation of E-cadherin [124]. Recently, it has been shown that dactolisib (PI3K/mTOR inhibitor) effectively treats *UBE2C*-driven orthotopic mouse xenografts of breast and lung cancer BM. Interestingly, early treatment with oral dactolisib prevents leptomeningeal dissemination in vivo [78].

A study on cervical carcinoma analyzed the structure, binding energy, chemical properties and drug-like properties of UBE2C and identified 2,4-diimino-1-methyl-1,3,5-triazepan-6-one, a sulfuric acid compound with 5,6-diamino-2,4-pyrimidinediol (1:1) and 7-alpha-d-ribofuranosyl-2-aminopurine-5’-phosphate as possible inhibitors [125]. Nonetheless, these candidates need additional experimental testing to prove their ability to inhibit UBE2C for therapeutic purposes. 

A novel in vitro biochemical assay that exploits the ability of UBE2C to ubiquitinate itself in particular conditions was developed to study the UBE2C ubiquitin-conjugating activity. This platform provides a rapid and sensitive time-resolved fluorescence resonance energy transfer-based assay that might be used to identify UBE2C inhibitors and can be scalable into an automated high-throughput screening layout [113]. Furthermore, the recently established patient-derived models of BM, which replicate human metastatic disease, can be used as platforms for the preclinical evaluation of anticancer therapies [19]. Both strategies can be applied to identify and validate novel UBE2C inhibitors for the treatment of brain cancer patients.

## 5. Discussion (And Future Directions)

Despite being a rare condition, malignant brain tumors cause a dismal prognosis and reduced life expectancy. Invasion and dissemination of cancer cells into the brain are important features of aggressiveness in brain cancer that urgently need a better therapeutic intervention aiming to improve patient prognosis [126].

UBE2C is a key enzyme during the cell cycle and has been considered a proto-oncogene and a tumor biomarker. High expression of UBE2C is observed in several different types of cancer and correlates with histological grade, poor prognosis, resistance to therapy, and relapse. Moreover, there is evidence of the role of UBE2C in proliferation, EMT, invasion, and migration in several malignancies, including brain tumors. 

Due to its characteristics, UBE2C has the potential to be used as a diagnostic and prognostic biomarker, including in liquid biopsies from cancer patients. However, most studies reporting the role of UBE2C in brain tumors are relatively recent, meaning this is an understudied subject. Particularly, an in-depth focus on the relevance of UBE2C in the tumor microenvironment is lacking to understand the best clinical strategy in UBE2C-driven carcinogenesis.

UBE2C can also be considered a promising therapeutic target. Several reports have highlighted the interaction between UBE2C and the PI3K/AKT/mTOR signaling pathway in driving tumor progression and metastases through a yet unknown mechanism. The evidence of efficacy from diverse PI3K/AKT/mTOR inhibitors in UBE2C downregulation and EMT reversion sheds light on possible novel therapeutic options. However, although genetic silencing and indirect inhibition have been successfully tested, there is still an unmet need to identify UBE2C-specific inhibitors with therapeutic efficacy. One of the most prominent challenges consists of the three-dimensional structure of E2 enzymes. The catalytic site of these enzymes is located in a region that unlikely allows specific interactions with small molecules with high affinity [34]. Moreover, the inhibition of the catalytic center can lead to the inhibition of other enzymes that bind ubiquitin. Another inhibitory strategy would be to prevent the binding of UBE2C to the APC/C complex, but the resolution of the APC/C-UBE2C complex is very low, making in silico drug screening a challenge. Lastly, the treatment of brain tumors implies the development of drugs that can cross the blood–brain barrier, which has been proven to be a demanding task.

In conclusion, despite the established relevance of UBE2C in cancer, its role in brain tumors should be further investigated to assess its potential value as a diagnostic and prognostic marker, as well as a potential therapeutic target.

## Figures and Tables

**Table 1:** Summary of UBE2C’s role and impact in different types of malignancies.

Malignancy Type	Synthesized Outcomes	References
*Breast cancer*	*UBE2C* overexpression is closely associated with high histological grade, lymphovascular invasion, larger tumors, HER2 positivity, early metastasis, increased mortality rates, and worse prognosis. Higher UBE2C levels are positively correlated with N-cadherin, matrix metalloproteinases, and cell cycle-related biomarkers (such as p53, Ki67, PI3K, and EGFR) while being negatively related to E-cadherin. ALKBH5 promotes breast cancer cell growth, stemness, and metastasis through the UBE2C/p53 axis. *UBE2C* knockdown increases PTEN levels and decreases p-AKT, p-mTOR, and HIF-1α levels, declining proliferation and invasion ability through the impairment of the AKT/mTOR signaling pathway. *UBE2C* knockdown sensitizes breast cancer cells to radiation and chemotherapy. UBE2C is correlated with CTLA4 expression.	[55,56,57,58,59,60,61]
*Lung cancer*	High UBE2C expression is associated with high histological grade, sex, TNM stage, age, angiogenesis, post-operative survival time, and poor prognosis in NSLCL. *UBE2C* knockdown inhibited NSLCL cell proliferation and increased chemotherapeutical sensitivity. *UBE2C* overexpression may play an important role in lung cancer EMT, invasion, migration, and metastasis. UBE2C expression is correlated with the p53 and EGFR mutational status. The UBE2C/CDH1/DEPTOR axis regulates cell cycle progression and autophagy in NSCLC.	[62,63,64,65,66,67]
*Hepatocellular carcinoma*	*UBE2C* overexpression is associated with high histological grade, p53 mutation, and poor survival. *UBE2C* expression is increased in sorafenib-resistant HepG2 cells. *UBE2C* silencing represses proliferation and colony formation of MHCC97H cells and overexpression enhances aggressiveness. UBE2C levels are positively associated with regulatory T cells and TFH infiltration and negatively correlated with infiltration of monocytes. *UBE2C* depletion sensitizes cells to chemotherapeutical drugs.	[68,69,70]
*Endometrial carcinoma*	*UBE2C* is upregulated in endometrial cancer cell lines and patients and is associated with high histological grade, worse subtypes, frequent recurrence, shorter overall survival, and poor outcome. *UBE2C* knockdown upregulates E-cadherin and downregulates vimentin, leading to a reduction in proliferation, invasion, and migration in RL95-2 and Ishikawa cells. UBE2C promotes EMT by negative p53 modulation.	[71]
*Thyroid carcinoma*	UBE2C is strongly associated with immune response. *UBE2C* knockdown decreases cell proliferation, migration, and invasion, promotes apoptosis, and reduces chemotherapeutic resistance.	[72]
*Adrenocortical/ clear cell renal carcinoma*	UBE2C expression is correlated with advanced tumor stage, high histological grade, and poor prognosis in renal carcinoma. *UBE2C* overexpression induces m^6^A methylation and promotes self-renewal of stem cells in adrenocortical carcinoma. *UBE2C* knockdown reduces proliferation, invasion, and migration, diminishes DNA damage repair, and induces apoptosis through cell cycle and EMT inhibition in adrenocortical carcinoma. UBE2C is strongly correlated with stromal score, immune score, and infiltration of M0 macrophages, regulatory T cells, and CD4+ memory T cells in clear cell renal carcinoma.	[73,74]
*Brain tumors*	UBE2C overexpression is associated with high histological grade, decreased overall survival, and poor prognosis in glioma. *UBE2C* expression is related to FoxM1 and AURKB levels. FoxM1 binds to the *UBE2C* promoter, inducing its transcription. *UBE2C* silencing induces autophagy, inhibits cell viability, promotes cell apoptosis, and promotes the activation of p53 in glioma cells. *UBE2C* knockdown inhibits the Akt-mTOR signaling pathway in glioma cells. Enriched UBE2C expression is associated with resistance to temozolomide and radiotherapy in glioma. *UBE2C* is highly expressed in brain metastasis from different origins and is associated with declined survival and leptomeningeal dissemination. Dactolisib (PI3K/mTOR inhibitor) treats UBE2C-driven breast and lung cancer brain metastasis, and early treatment prevents leptomeningeal dissemination in vivo.	[75,76,77,78]

Abbreviations. UBE2C: ubiquitin-conjugating enzyme, HER2: human epidermal growth factor receptor 2, p53: tumor suppressor p53, Ki67: marker of proliferation Ki-67, PI3K: phosphoinositide 3-kinase, EGFR: epidermal growth factor receptor, HIF-1α: hypoxia-inducible factor 1-alpha, NSCLC: non-small cell lung carcinomas, TNM: TNM Classification of Malignant Tumors, AKT: AKT serine/threonine kinase, CTLA4: cytotoxic T-lymphocyte-associated protein 4, EMT: epithelial–mesenchymal transition, TFH: T follicular helper cells, FoxM1: Forkhead box M1, AURKB: aurora kinase B, mTOR: mammalian target of rapamycin.

## Data Availability

No new data were created or analyzed in this study. Data sharing is not applicable to this article.
